# Genome-Wide Identification and Expression Analysis of the *TGA* Gene Family in Banana (*Musa nana Lour.*) Under Various Nitrogen Conditions

**DOI:** 10.3390/ijms26052168

**Published:** 2025-02-28

**Authors:** Bencheng Zhang, Wei Wang, Can Wang, Bingyu Cai, Junting Feng, Dengbo Zhou, Yufeng Chen, Miaoyi Zhang, Dengfeng Qi, Zhuo Wang, Yongzan Wei, Jianghui Xie

**Affiliations:** 1 National Key Laboratory for Tropical Crop Breeding, School of Tropical Agriculture and Forestry, Hainan University, Sanya 570228, China; 22220951310218@hainanu.edu.cn (B.Z.); canw0131@163.com (C.W.); 2 National Key Laboratory for Tropical Crop Breeding, Institute of Tropical Bioscience and Biotechnology, Chinese Academy of Tropical Agricultural Sciences, Haikou 571101, China; wangwei@itbb.org.cn (W.W.); cai_by15607582585@163.com (B.C.); fengjunting@itbb.org.cn (J.F.); zhoudengbo@itbb.org.cn (D.Z.); chenyufeng@itbb.org.cn (Y.C.); zhangmiaoyi@itbb.org.cn (M.Z.); qidengfeng@itbb.org.cn (D.Q.); wangzhuo@itbb.org.cn (Z.W.); 3 Sanya Research Academy, Chinese Academy of Tropical Agriculture Science, Sanya 572019, China

**Keywords:** banana, nutrient utilization, *TGA* transcription factors, gene expression

## Abstract

The *TGA* (TGACG motif-binding factor) transcription factors are integral to root growth and development, and are pivotal in mediating plant responses to abiotic stresses. Nonetheless, their role in the nutrient absorption processes of banana plants has not been extensively investigated. This research conducted a comprehensive analysis of the *MaTGA* gene family, emphasizing their physicochemical characteristics, phylogenetic relationships, gene duplication events, promoter cis-regulatory elements and protein interaction networks. Furthermore, this study investigated the expression patterns of *MaTGA* family members under varying nitrogen conditions. A total of 18 *MaTGA* members were identified within the banana genome, each encoding proteins characterized by the presence of bZIP and DOG domains. These genes exhibited an uneven distribution across eight chromosomes. Phylogenetic analysis further classified the *MaTGA* family into four distinct subgroups (I–IV), consisting of three, seven, three, and five members, respectively. An analysis of promoter cis-elements indicated that over 50% of the *MaTGA* gene family members contain hormone-responsive elements associated with abscisic acid (ABRE), ethylene (ERE), and salicylic acid (SARE), in addition to stress-responsive elements related to drought (MBS) and low temperature (LTR). Regarding gene expression, *MaTGA7*, *MaTGA8*, and *MaTGA15* exhibited significantly elevated expression levels in the leaves and roots relative to other tissues. Under varying nitrogen conditions, 13 members, including *MaTGA7* and *MaTGA8*, demonstrated the highest expression levels under reduced nitrogen (70%) treatment, followed by low nitrogen (20%) conditions, and the lowest expression levels were observed under nitrogen-deficient conditions. These findings imply that *MaTGA* genes may play crucial roles in enhancing nitrogen use efficiency. Protein interaction predictions suggest that MaTGA7, MaTGA8, and MaTGA15 may interact with nitrogen-related proteins, including Nitrate Transporter 2 (NRT2.1 and NRT2.2), NIN-Like Protein 7 (NLP7), and Nitrate Transporter 1.1 (NPF6.3). In summary, *MaTGA7*, *MaTGA8*, and *MaTGA15* are likely involved in the processes of nitrogen absorption and utilization in bananas. The present findings establish a basis for subsequent investigations into the functional roles of *MaTGA* genes in augmenting nutrient use efficiency and mediating responses to abiotic stresses in banana plants.

## 1. Introduction

The *Basic Leucine Zipper* (*bZIP*) transcription factor family is integral to essential biological processes, including plant development, hormone signaling, and responses to abiotic stressors [1,2]. Within this family, the TGACG Element Binding Factor (*TGA*) genes represent a subgroup of transcription factors that exhibit specificity in binding to TGACG/as-1 elements. These genes are distinguished by the presence of conserved bZIP and DOG domains and are categorized within group D of the *bZIP* family [3]. The initial identification and cloning of *TGA* genes were accomplished in tobacco (*Nicotiana tabacum* L.) [4]. Since their discovery, *TGA* transcription factors have been extensively investigated across various plant species, including Arabidopsis (*Arabidopsis thaliana* L.) [3], rice (*Oryza sativa* L.) [5], maize (*Zea mays* L.) [6], soybean (*Glycine max* L.) [7], and barley (*Hordeum vulgare* L.) [8].

These transcription factors are crucial for plant growth, development, and adaptation to abiotic stresses. In the model organism Arabidopsis (*A. thaliana*), *TGA* family members are particularly significant in regulating root growth and development [5]. For instance, *AtTGA3* has been shown to promote root development by mitigating cadmium (Cd) accumulation [9]. In the Attga1tga4 double mutant, there is a notable reduction in primary root length and lateral root formation, attributed to the regulatory role of *TGA* in the expression of nitrate transporter genes *AtNRT2.1* and *AtNRT2.2*, which are crucial for lateral root development [5,10]. Mutations in *TGA1*, *TGA3*, *TGA4*, and *TGA7* result in shorter roots and smaller differentiated cells, underscoring their synergistic contributions to root development [6]. In soybean, *GmTGA* genes are implicated in the formation of nodules and root hairs [11].

Furthermore, *TGA* transcription factors are integral to the modulation of plant morphology. In rice, the *TGA* transcription factor *OsbZIP49* modulates GH3-mediated auxin degradation through the regulation of *OsGH3-2* and *OsGH3-13* expression, which in turn diminishes gravitropic responses in the stems and influences tiller angles [12]. In maize, the transcriptional activity of the *TGA* transcription factor *FEA4* is modulated by the redox state of the GRX proteins, thereby affecting inflorescence meristem development and overall plant growth [13]. Similarly, in Arabidopsis, the transcription factors *AtTGA1*, *AtTGA4*, *AtTGA2*, *AtTGA5*, and *AtTGA6* govern leaf hyponasty in response to variations in light, temperature, and auxin levels [14]. *TGA* transcription factors are integral to the regulation of flowering. In Arabidopsis, *ROXY9* modulates plant height and flowering time through a mechanism dependent on *TGA1* and *TGA4* [15]. Specifically, *TGA4* interacts with *CONSTANS* (*CO*) to regulate the expression of *FLOWERING LOCUS T* (*FT*), thereby influencing flowering time [16]. Furthermore, mutations in *TGA7* result in delayed flowering by affecting the expression of the key flowering repressor gene *FLC* (*FLOWERING LOCUS C*) [17].

Additionally, *TGA* transcription factors play pivotal roles in plant responses to abiotic stresses, including low nitrogen, low phosphorus, salt stress, and drought. Abiotic stresses can perturb the ionic and hormonal equilibrium in plants, thereby impacting metabolic processes and impeding plant growth and development [18]. Under the conditions of nitrogen deficiency, transgenic *OE-TGA4* Arabidopsis plants exhibited superior leaf and root growth relative to wild-type Arabidopsis [19]. In apple, *TGA2.1* interacts with *MdSCL14* to form a complex that enhances the plant’s resistance to salt stress [20]. In soybean, *GmTGA13* facilitates the maintenance of normal growth under high salinity conditions by decreasing sodium ion uptake and augmenting potassium and calcium ion content [21]. *AtTGA4* facilitates nitrate transport and assimilation in Arabidopsis, thereby promoting root development and enhancing drought resistance [19]. In general, *TGA* transcription factors are crucial in enabling plants to endure abiotic stresses.

The banana (*Musa nana* Lour.), a member of the Musaceae family, is identified by the Food and Agriculture Organization (FAO) as the fourth most significant food crop in developing countries, following rice, wheat, and maize [22]. During their growth phase, banana plants necessitate substantial quantities of nutrients, including nitrogen, phosphorus, and potassium. This demand has led to the prevalent adoption of “high water and high fertilizer” cultivation practices. Nevertheless, the over-application of fertilizers contributes to considerable nutrient leaching and environmental contamination. Enhancing nitrogen use efficiency in banana cultivation presents a viable solution to mitigate these adverse effects. Therefore, it is imperative to identify key genes involved in nitrogen utilization and to develop banana varieties with improved nitrogen efficiency. This study conducted a comprehensive genome-wide identification and bioinformatics analysis of the *TGA* gene family in bananas, examining the expression patterns of the *TGA* genes across various tissues and under different nitrogen conditions through quantitative real-time PCR (qRT-PCR). The results offer an initial screening of the *TGA* genes potentially involved in nitrogen absorption, thereby establishing a foundation for future investigations into the functional roles of *TGA* genes and the molecular mechanisms governing nitrogen utilization in bananas.

## 2. Results

### 2.1. Analysis of the Physicochemical Properties and Phromosomal Pocalization of TGA Gene Family Members in Banana

Utilizing the banana genome database and employing *AtTGA* as a reference framework, a comprehensive genome-wide prediction of the *MaTGA* gene family was conducted through BLAST (https://blast.ncbi.nlm.nih.gov/Blast.cgi (accessed on 1 December 2024) alignment [23]. This analysis led to the identification of 18 candidate TGA genes, which were designated as *MaTGA1* to *MaTGA18*, according to their chromosomal locations and sequences.

The physicochemical characteristics of these 18 MaTGA transcription factor family members were subsequently examined. The amino acid sequence lengths of these genes varied from 332 amino acids (*MaTGA6/9*) to 509 amino acids (*MaTGA14*), with molecular weights ranging from 37.05 kDa (*MaTGA6*) to 56.69 kDa (*MaTGA14*). The predicted theoretical isoelectric points of the MaTGA proteins varied between 5.83 (MaTGA3) and 9.06 (*MaTGA10*). Subsequent analysis demonstrated that all MaTGA proteins exhibited instability indices exceeding 40, suggesting a relative instability [24]. The aliphatic index ranged from 73.23 (*MaTGA16*) to 86.97 (*MaTGA11*) [25], while the hydrophilicity index for all proteins was negative, indicating their hydrophilic nature [26]. Predictions regarding subcellular localization suggested that the MaTGA proteins are situated in the nucleus, aligning with their function as transcription factors (Table 1).

**Figure 1:**
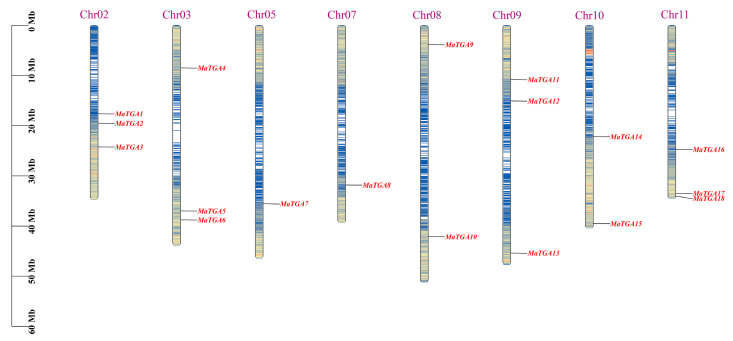
Distribution of the 18 *MaTGA* genes on the chromosomes of the banana. Chr: chromosome. The left scale indicates chromosome length in megabytes (Mb).

Utilizing comprehensive gene localization data, the 18 *MaTGA* genes were mapped across eight chromosomes within the banana genome, specifically on chromosomes Chr02, Chr03, Chr05, and Chr07 through to Chr11. Notably, chromosomes Chr02, Chr03, Chr09, and Chr11 each harbored three *TGA* genes, whereas chromosomes Chr08 and Chr10 each contained two *TGA* genes. In contrast, chromosomes Chr05 and Chr07 each possessed a single *TGA* gene. This distribution pattern indicates a non-uniform and irregular arrangement of the *MaTGA* genes across the chromosomes (Figure 1).

### 2.2. Phylogenetic Analysis of MaTGA Family Genes

**Figure 2:**
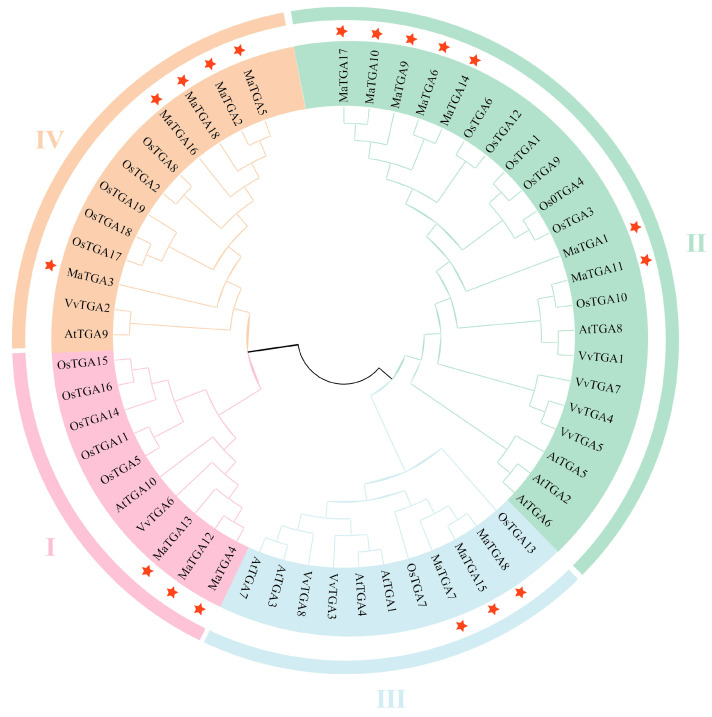
Phylogenetic relationship of the *MaTGA* family genes. The four groups (I to IV) are represented by different colors, and *MaTGA* members are represented by stars.

To examine the classification and phylogenetic relationships within the *MaTGA* gene family, a comprehensive phylogenetic analysis was conducted. This analysis incorporated *MaTGA* genes (18) from banana, *AtTGA* genes (10) from Arabidopsis, *OsTGA* genes (19) from rice, and *VvTGA* genes (8) from grape. The phylogenetic tree was constructed utilizing the Neighbor-Joining (NJ) method implemented in MEGA7.0 software. The clustering analysis results indicated that the *MaTGA* genes from banana are categorized into four distinct subfamilies (I–IV). Subfamily II comprised the largest number of members, encompassing seven *MaTGA* genes, which clustered alongside four *AtTGA* genes from *Arabidopsis*, seven *OsTGA* genes from rice, and four *VvTGA* genes from grape. Subfamily IV included five *MaTGA* genes, whereas subfamilies I and III each contained three *MaTGA* genes. Of particular interest, the *MaTGA* genes *MaTGA7*, *MaTGA8*, and *MaTGA15* from subfamily III clustered with *AtTGA1* and *AtTGA4* from *Arabidopsis*, indicating a potential role in root development and nutrient absorption in banana (Figure 2).

### 2.3. Conserved Domain and Motif Analysis of the MaTGA Family Genes

**Figure 3:**
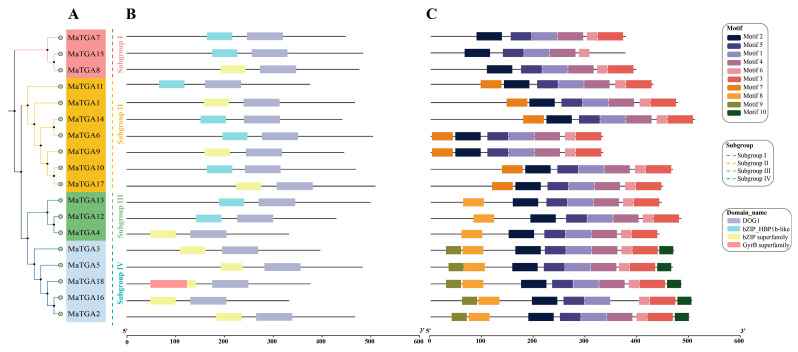
Conserved domains and motifs of the *MaTGA* members. (**A**) Phylogenetic tree; (**B**) Conserved domain; (**C**) Conserved motif.

To explore the potential functions of the *MaTGA* family members in bananas, we conducted an analysis of the conserved domains within the *MaTGA* family. Our findings indicated that all *MaTGA* family members possess the bZIP and DOG1 domains, which are characteristic of the *TGA* family. Additionally, we employed the MEME suite to examine the distribution of 10 key motifs within the TGA protein sequences of the banana. The analysis revealed that all 18 *MaTGA* genes contain motifs 1, 2, 5, and 6, suggesting these motifs may represent common features of the *MaTGA* gene family (Figure 3 and Figure S1).

With the exception of *MaTGA15* in subfamily I, all other members of the *MaTGA* family possess motif 3. Variations in motif composition were identified among the subfamilies. Beyond the shared motifs, members of subfamily II also exhibit motif 7, whereas subfamilies III and IV both incorporate motif 8. Notably, subfamily IV uniquely harbors motif 9 and 10. These differences in motif distribution may be associated with the distinct biological function characteristic of each subfamily.

### 2.4. MaTGA Genes Duplication Event Analysis

**Figure 4:**
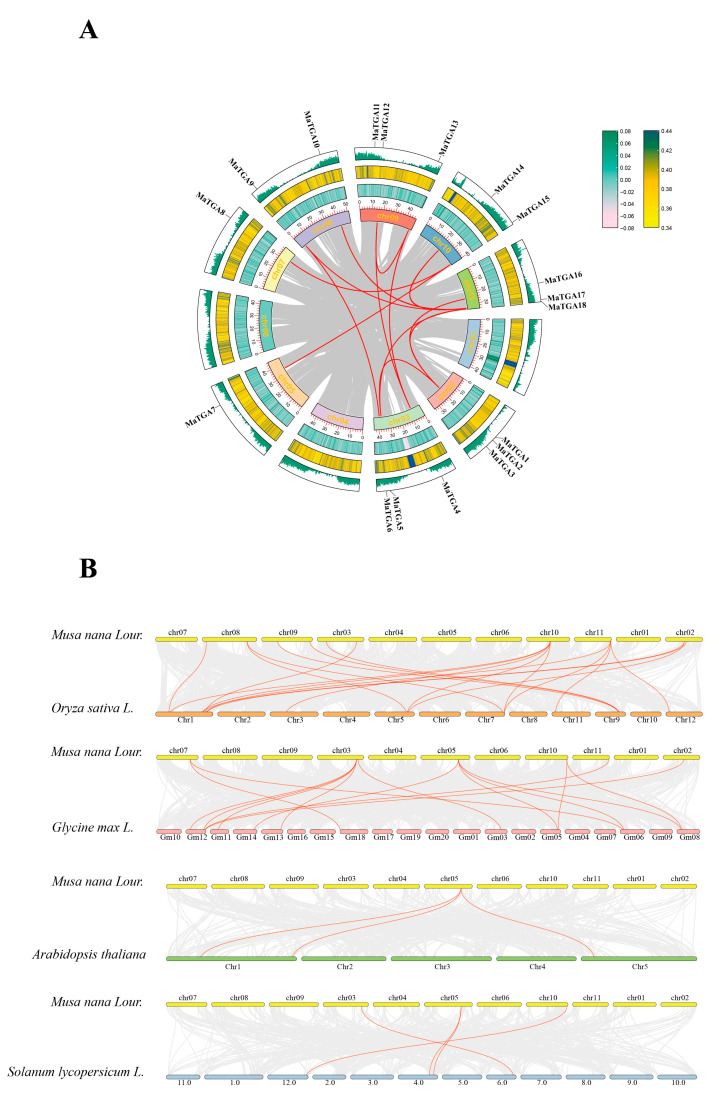
Intergenomic and collinear relationships of the *MaTGA* family. (**A**) The diagram illustrates, from the innermost to the outermost layer, gene chromosome localization, GC skew, GC ratio, and gene density. (**B**) The interspecific collinearity analysis is presented for rice (*O. sativa*), soybean (*G*. *max*), Arabidopsis *(A. thaliana*), and tomato (*S. lycopersicum*), arranged from top to bottom. The gray line represents the collinearity of all the genes in the banana, and the red line represents the collinearity of the *MaTGA* genes.

To examine the origin, expansion, and diversification of the *TGA* gene family, and to explore its evolutionary history, functional diversity, and regulatory mechanisms, we conducted an analysis of the copy number, distribution patterns, and gene duplication events of the *TGA* family members in banana and other species (Figure 4A). The synteny analysis revealed 13 collinear gene pairs within the *MaTGA* family, including *MaTGA2*–*MaTGA5*, *MaTGA2*–*MaTGA16*, *MaTGA2*–*MaTGA18*, *MaTGA6*–*MaTGA9*, *MaTGA4*–*MaTGA12*, *MaTGA4*–*MaTGA13*, *MaTGA5*–*MaTGA18*, *MaTGA7*–*MaTGA15*, *MaTGA8*–*MaTGA15*, *MaTGA9*–*MaTGA17*, *MaTGA10*–*MaTGA17*, *MaTGA12*–*MaTGA13*, and *MaTGA14*–*MaTGA17*. The observed collinearity relationships were attributed exclusively to segmental duplication events, as no tandem duplications were detected [27].

To further investigate the evolutionary history and phylogenetic relationships of the *TGA* genes across different species, we performed an interspecies synteny analysis involving rice, soybean, Arabidopsis, and tomato. The analysis revealed that the numbers of collinear genes between the banana *TGA* genes and those in rice, soybean, Arabidopsis, and tomato were nineteen, sixteen, three, and four, respectively (Figure 4B). Banana and rice, both classified as monocotyledons, demonstrated the highest number of collinear genes, significantly exceeding those observed in dicotyledons such as Arabidopsis (*A. thaliana*) and tomato (*S. lycopersicum*). Remarkably, the 16 collinear genes shared between banana and soybean (*G. max*), another dicotyledon, closely approximate the number shared with rice (*O. sativa*). This observation implies that the genomes of banana and soybean may have experienced more extensive gene family duplication events, resulting in a greater number of collinear genes and potentially more analogous functions.

### 2.5. Prediction and Analysis of the MaTGA Protein Structures

In order to examine the folding and stability of the MaTGA family of proteins and to elucidate the role of secondary structures in biological processes such as signal transduction and metabolic catalysis, we conducted a predictive analysis of the secondary structures of the MaTGA family members. The results of this prediction indicated that all 18 MaTGA proteins possess α-helices, β-turns, extended strands, and random coils (Table 2). Within this study, the α-helix content was observed to range from 49.79% to 74.70%, and the random coil content varied between 17.17% and 41.12%, collectively constituting the predominant components of the protein structure. Conversely, the β-turn and extended chain elements were present in comparatively minor proportions, with β-turns ranging from 1.34% to 3.37% and extended chains from 4.83% to 8.53% (Table 2). The predominance of α-helices and random coils in the MaTGA proteins imply that these structural elements are instrumental in mediating a balance between functional execution and stability, thereby facilitating the protein’s specific biological functions. Conversely, the relatively lower abundance of β-turns and extended chains may suggest a secondary role in the overall structural configuration.

### 2.6. Analysis of the MaTGA Promoter cis-Acting Elements

**Figure 5:**
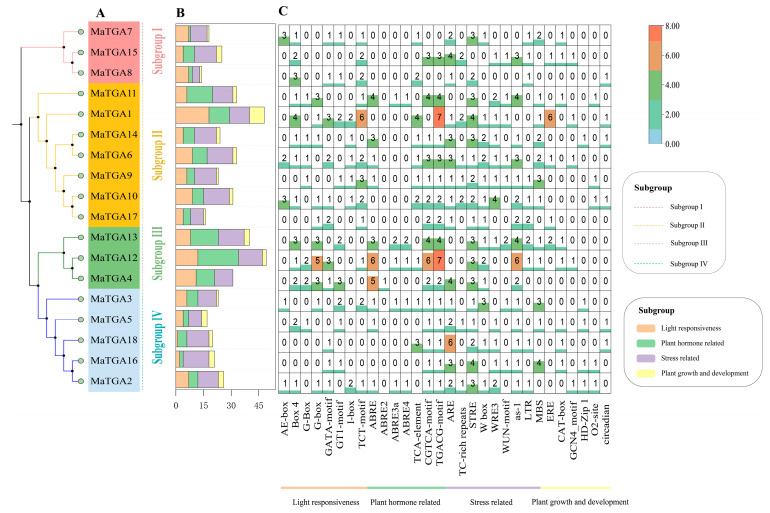
Analysis of the *MaTGA* promoter cis-acting elements. (**A**) Phylogenetic tree; (**B**) Statistical histogram of the four types of responsive elements; (**C**) Heat map of the number of promoter cis-acting elements.

Various promoter cis-acting elements are typically linked to distinct biological functions. To investigate the biological functions of the *MaTGA* genes, an analysis of its promoter cis-acting elements was conducted. The predictive analysis of these elements indicated that the cis-acting elements of all *MaTGA* genes could be predominantly classified into four categories: light response elements, plant growth and development response elements, hormone response elements, and stress response elements (Figure 5). The optical response elements comprised eight distinct types (AE-box, Box 4 and GT1-motif). In contrast, the elements associated with plant growth and development encompassed 11 categories, which include those involved in the regulation of circadian rhythms (circadian), seed-specific regulation (RY-element), and the differentiation of mesophyll cells within the leaf (HD-Zip 1).

Furthermore, an analysis revealed the presence of 15 distinct types of hormone response elements, with more than 50% of the *MaTGA* exhibiting abscisic acid response elements (ABRE, ABRE2, ABRE3a, ABRE4), ethylene response elements (ERE), auxin response elements (AuxRR-core, TGA-box, TGA-element), gibberellin response elements (GARE-motif, P-box, TATC-box), and salicylic acid response elements (SARE, TCA-element). Notably, all *MaTGA* genes, with the exception of *MaTGA7* and *MaTGA8*, contain methyl jasmonate response elements (CGTCA-motif, TGACG-motif). The TGA promoter sequences encompassed six distinct types of stress response elements, namely drought-responsive elements (MBS), low-temperature-responsive elements (LTR), hypoxia-specific inducible response elements (GC-motif), along with defense and stress response elements such as TC-rich repeats, W box, and WUN-motif (Figure 5). These findings suggest that *MaTGA* genes are likely to play a pivotal role in the growth and development of banana plants, in addition to their involvement in hormone responses and stress tolerance.

### 2.7. Expression Characteristics of the MaTGA Genes in Different Parts of the Banana Plant

**Figure 6:**
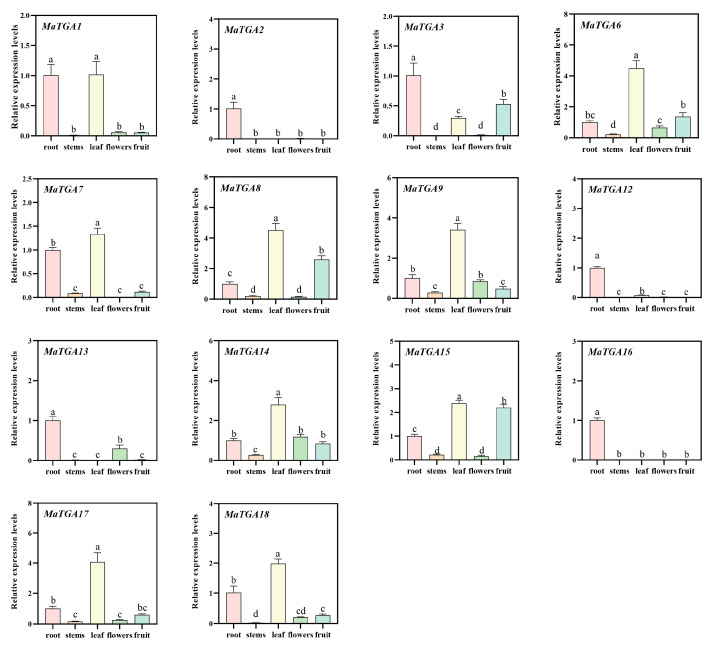
Gene expression profiles of the *MaTGA* family in the different tissue (root, stem, leaf, flower, fruit) parts of the banana. Statistical significance was defined as *p* < 0.05. The notation “a and a” signifies that the difference is not statistically significant, whereas “a and b” denotes a statistically significant difference.

To examine the tissue-specific expression of the *MaTGA* genes in bananas, this study conducted an analysis of the expression profiles of the *MaTGA* genes across various tissues of the baxijiao (Cavendish) banana, encompassing the roots, pseudostems, leaves, flowers, and fruits. Quantitative real-time PCR (qRT-PCR) results indicated that *MaTGA1* and *MaTGA7* demonstrated elevated expression levels in the roots and leaves, whereas *MaTGA3* exhibited significantly higher expression in the roots relative to other tissues. The expression levels of *MaTGA6*, *MaTGA9*, *MaTGA14*, *MaTGA17*, and *MaTGA18* were markedly elevated in the leaves relative to other tissues. Additionally, in comparison to the root, stem, and flower, the expression levels of *MaTGA6*, *MaTGA8*, and *MaTGA15* were also higher in the fruit. The expression levels of *MaTGA2*, *MaTGA12*, and *MaTGA16* were high in the root system, but hardly in other tissues. *MaTGA13* was only expressed in the roots and flowers, but its expression level was low in flowers (Figure 6).

### 2.8. qRT-PCR Verified the Expression Patterns of the MaTGA Gene Family at Different Nitrogen Levels

**Figure 7:**
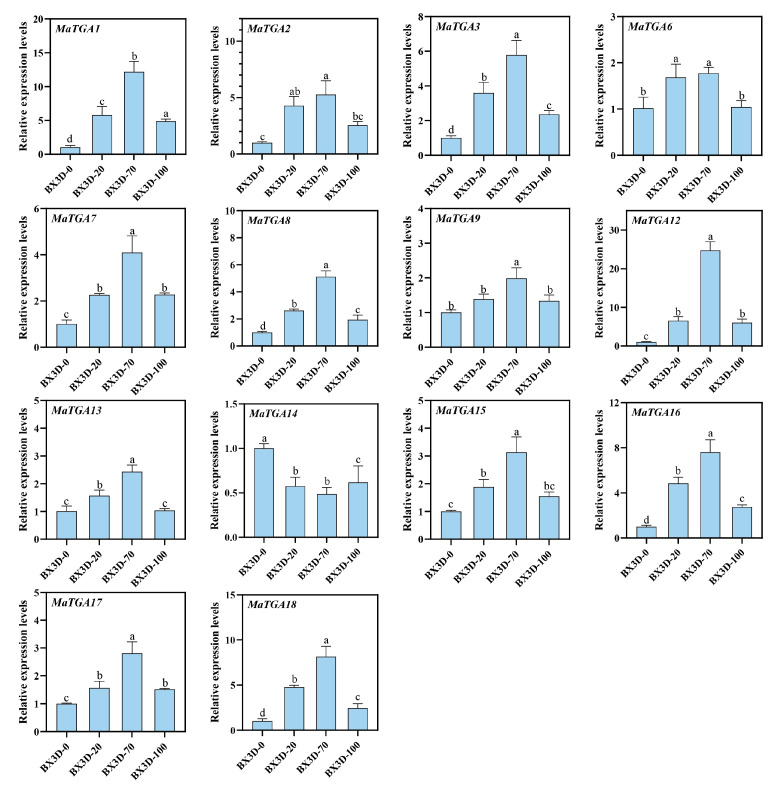
Gene expression profiles of the *MaTGA* family, in response to different nitrogen levels (3 days: 0 N, 20% N, 70% N, 100% N). BX3D-0: No nitrogen treatment for 3 days, BX3D-20: 20% nitrogen treatment for 3 days, BX3D-70: 70% nitrogen treatment for 3 days, BX3D-100: 100% nitrogen treatment for 3 days. Statistical significance was defined as *p* < 0.05. The notation “a and a” signifies that the difference is not statistically significant, whereas “a and b” denotes a statistically significant difference.

The *TGA* gene is integral to the plant’s response to nitrogen availability and the regulation of nitrate uptake, as indicated by previous studies [28]. To investigate this further, the expression patterns of the *TGA* genes in the roots were analyzed under varying nitrogen levels (100%, 70%, 20%, and 0%), utilizing qRT-PCR (Figure 7). Except for *MaTGA14*, the expression patterns of *MaTGA1*, *MaTGA2*, *MaTGA3*, *MaTGA7*, *MaTGA8*, *MaTGA9*, *MaTGA12*, *MaTGA13*, *MaTGA15*, *MaTGA16*, *MaTGA17*, and *MaTGA18* were highly similar. Under the reduced nitrogen treatment (70%), these genes exhibited the highest expression levels, which decreased under low nitrogen conditions (20%), and were lowest in the absence of nitrogen (Figure 7). This pattern indicates that several members of the *MaTGA* family are likely implicated in the response to and uptake of varying nitrogen levels in banana roots, with their functions demonstrating both similarity and potential synergistic interactions.

### 2.9. MaTGA Protein–Protein Interaction (PPI) Prediction Analysis

**Figure 8:**
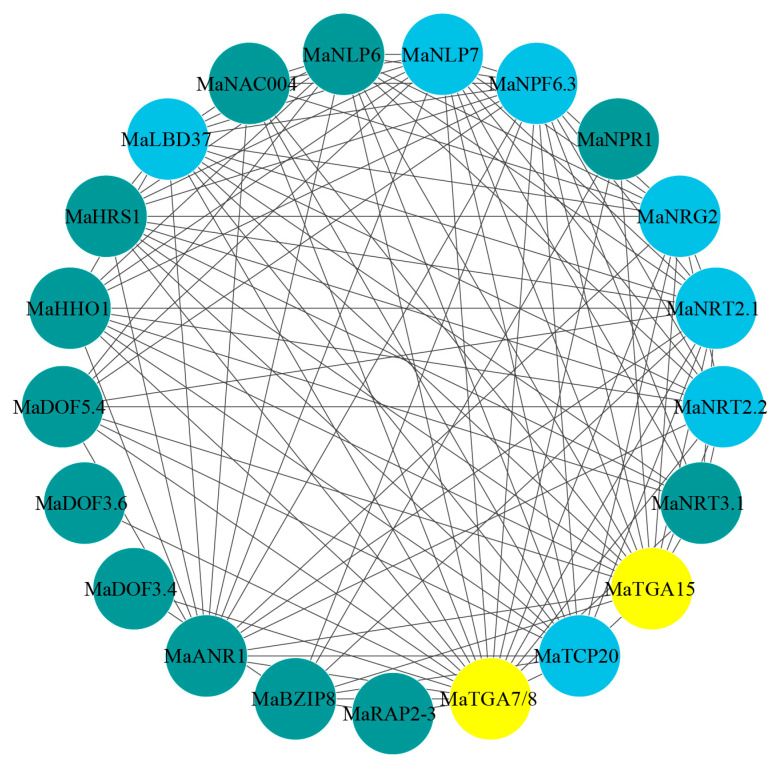
MaTGA7/8/15 protein interaction prediction. The gray lines represent possible interactions between the protein. Yellow circles represent MaTGA proteins, blue circles represent nitrogen-related proteins that interact with MaTGA, and cyan circles represent other proteins that interact with MaTGA.

Protein–protein interactions constitute intricate networks essential for various biological processes, including signal transduction, metabolic regulation, and the cell cycle. Predicting the interaction network of the MaTGA family members can provide insights into the collaborative mechanisms by which the MaTGA proteins execute biological functions. Protein interaction predictions indicated that MaTGA7/8/15 may interact with several pivotal proteins implicated in nitrogen absorption and regulation (Figure 8). Notably, these interactions involve several key genes. For instance, NRT2.1 (Nitrate Transporter 2.1) [29] and NRT2.2 (Nitrate Transporter 2.2) [30] are important. The major nitrate signaling regulator, NIN - Like Protein 7 (NLP7) [31], also plays a significant role. Nitrate Transporter 1.1 (NPF6.3), which is crucial for nitrate transport [32], is part of these interactions. Additionally, regulatory genes like Teosinte Branched1/Cycloidea/Proliferating Cell Factor 20 (TCP20) [33], which respond to both nitrate and auxin signaling, are integral to nitrate signaling pathways. Furthermore, Lateral Organ Boundaries Domain 37 (LBD37), a negative regulator of plant nitrogen metabolism [34], and Nitrate Reductase Gene 2 (NRG2), which activates the expression of nitrate-responsive genes like NRT1.1 and NRT2.1, contribute significantly to the enhancement of nitrate absorption and metabolism [35]. MaTGA7/8/15 are likely to play important roles in the nitrogen absorption and signaling network in bananas, with their functions potentially mediated through interactions with key regulatory genes (Figure 8).

## 3. Discussion

*TGA* (TGACG-binding) transcription factors, belonging to the *bZIP* D family, are integral to plant growth, development, and stress response mechanisms. These factors exert their regulatory functions by binding to the TGACG sequence, also referred to as activation sequence 1 (as-1), thereby modulating genes associated with plant immunity and hormone signaling pathways [36]. In this study, a total of 18 *MaTGA* genes were identified through homologous sequence comparison and conserved domain screening, utilizing the *AtTGA* family protein sequences as reference models, based on the banana reference genome data. This number is comparable to the 19 *OsTGA* genes found in rice, and exceeds the number identified in dicotyledonous plants, such as the *AtTGA* in *Arabidopsis* (10) and the *VvTGA* (8) in grape [11,37,38]. Phylogenetic analysis indicated that the 18 *MaTGA* genes in banana can be categorized into four distinct subfamilies. Members of the *MaTGA* gene family within the same subfamily exhibited conserved domains and motifs, implying that these genes are conserved across various species. *MaTGA7*, *MaTGA8*, and *MaTGA15* were found to be members of the same subfamily as *AtTGA1* and *AtTGA4* in the model organism Arabidopsis thaliana and are posited to play a role in nitrate transport and assimilation within root structures. Typically, gene duplication events serve as the principal mechanism facilitating the expansion of gene families [39]. Within the *MaTGA* family, 13 instances of fragment duplication were identified, although there was no indication of replication processes such as tandem duplication. The interspecies collinearity analysis indicated that the numbers of TGA genes in banana (*Musa nana* Lour.), rice (*Oryza sativa* L.), soybean (*Glycine max* L.), Arabidopsis (*Arabidopsis thaliana* L.), and tomato (*Solanum lycopersicum* L.) were eighteen, nineteen, sixteen, three, and four, respectively (Figure 4B). The *TGA* genes in banana and rice, both monocotyledonous species, exhibited a closer phylogenetic relationship and were more numerous compared to those in dicotyledonous species such as Arabidopsis and tomato. Notably, the *MaTGA* gene family also included several homologous genes to those found in the dicotyledonous soybean, which are integral to nitrogen utilization and fixation [40]. Given this similarity, it is hypothesized that the *MaTGA* genes in bananas may play a role in regulating nitrogen uptake, thereby providing insights into the molecular mechanisms underlying efficient nitrogen utilization in this species. Promoter cis-acting elements, situated in the promoter regions of genes, are affected by factors such as light, hormones, and stress. These elements interact with transcriptional regulatory factors, thus modulating the expression of specific genes [41]. The *MaTGA* promoter sequence encompassed a diverse array of cis-acting elements, notably including eight critical light-responsive elements, eleven elements associated with plant growth and development, fifteen hormone-responsive elements, and six elements related to defense and stress responses. These findings imply that *MaTGA* genes may have a significant role in banana growth and development, hormone regulation, and stress responses, such as those induced by drought and salinity. This provides a theoretical foundation for investigating the potential functions of *MaTGA* genes in stress resistance and growth regulation in bananas [28,42]. In terms of the expression characteristics in different tissues, *MaTGA1* and *MaTGA7* showed high expression levels in the roots and leaves, *MaTGA2*, *MaTGA12*, and *MaTGA16* were highly expressed in the roots, but were hardly expressed in other tissue parts, and *MaTGA13* was only expressed in the roots and flowers, where the expression level in flowers was also low. These results indicate that they may play an important role in nitrogen absorption, transport, and assimilation in bananas. Conversely, *MaTGA6*, *MaTGA8*, *MaTGA9*, *MaTGA14*, *MaTGA15*, *MaTGA17*, and *MaTGA18* exhibited significantly higher expression levels in the leaves compared to other tissues. This observation implies that these genes may be crucial for photosynthesis, growth, and development, as well as environmental adaptability, such as drought resistance, in bananas [43,44]. The findings presented herein offer significant insights into the role of the *MaTGA* genes in the growth and development of bananas.

Members of the *TGA* family are integral to root growth and the formation of root hairs, as evidenced by previous studies [5,10,11]. In this study, with the exception of *MaTGA14*, the expression levels of *MaTGA1*, *MaTGA2*, *MaTGA3*, *MaTGA6*, *MaTGA7*, *MaTGA8*, *MaTGA9*, *MaTGA12*, *MaTGA13*, *MaTGA15*, *MaTGA16*, *MaTGA17*, and *MaTGA18* were observed to be highest under the conditions of nitrogen reduction (70%), followed by low nitrogen (20%), and were lowest in the absence of nitrogen (Figure 7). These findings indicate that *MaTGA* genes may play a crucial role in enhancing nitrogen use efficiency. Subsequent predictions of the MaTGA protein interaction network suggest that MaTGA7/8/15 may interact with proteins associated with nitrogen absorption and transport, including NRT2.1, NRT2.2, NLP7, and NPF6.3 (Figure 8). This implies that MaTGA7/8/15 could regulate the expression of nitrate-responsive genes through its interaction with NLP7, thereby facilitating the dynamic optimization of nitrogen uptake efficiency in plants under fluctuating nitrate availability. Moreover, MaTGA7/8/15 is likely to interact with proteins including TCP20, LBD37, and NRG2, which may facilitate the expression of genes associated with nitrate uptake, such as NRT1.1 and NRT2.1, by participating in the functional modules of LBD37 and NRG2. Additionally, MaTGA7/8/15 may regulate NPF6.3 (also known as NRT1.5) to mediate the long-distance transport and distribution of nitrate, thereby ensuring the nitrogen demands of high-nitrogen crops are satisfied [45]. The interaction with TCP20 indicates a potential linkage between nitrate and auxin signaling pathways, suggesting a pivotal role in the coordination of nitrogen uptake and root development. MaTGA7/8/15 may augment plant adaptability to low nitrogen stress through its interactions with bZIP8 and other stress-related factors. Under the conditions of nitrogen stress, *MaTGA7/8/15* are capable of activating the expression of critical genes, thereby enhancing nitrogen absorption capacity and alleviating the adverse effects of stress on plant growth. These findings indicate that *MaTGA7/8/15* is integral to the regulation of nitrogen metabolism and the enhancement of stress resistance, providing a novel perspective for investigating the mechanisms underlying nitrogen utilization and stress tolerance in bananas.

## 4. Materials and Methods

### 4.1. Plant Materials and Data Sources

Baxijiao (Cavendish) banana seedlings, utilized for the investigation of MaTGA tissue expression specificity, were cultivated at the Danzhou District base of the Chinese Academy of Tropical Agricultural Sciences. In contrast, those employed to examine MaTGA expression patterns under varying nitrogen conditions were cultivated at the National Key Laboratory for Tropical Crop Breeding.

For the study of tissue-specific expression, the baxijiao (Cavendish) banana was selected as the focal subject. Samples were collected from the root, stem, leaf, flower, and fruit of the plants, immediately cryopreserved in liquid nitrogen, and stored at −80 °C for further analysis. During the nitrogen treatment experiment, vigorous tissue-cultured ‘Brazilian’ banana seedlings were transplanted into cylindrical pots measuring 13 cm in diameter and 11 cm in height, which were filled with sterile, washed sand. The nitrogen concentration that supported the standard growth of banana seedlings was designated as the control. Four distinct nitrogen concentrations were applied: normal nitrogen (16 mM), reduced nitrogen (11.2 mM), low nitrogen (3.2 mM), and no nitrogen (0 mM). Each treatment comprised 50 replicates, with each plant receiving 300 mL of nutrient solution containing the specified nitrogen concentration on a weekly basis. Subsequently after the treatment, samples from the roots, stems, and leaves of the banana seedlings were collected at time intervals of 0 h, 24 h, 3 days, 7 days, 14 days, and 28 days. The samples were rapidly frozen using liquid nitrogen and subsequently stored at −80 °C for further analysis. Each experimental treatment was performed in triplicate.

### 4.2. Genome-Wide Identification of the TGA Genes in Banana

The genomic data for bananas, encompassing gene files (Musa_acuminata_pahang_v4.), coding DNA sequences (CDS), protein data, and GFF annotation files, were procured from the Banana Genome Database (https://banana-genome-hub.southgreen.fr/organism/1?tripal_pane=group_data (accessed on 1 October 2024)) [46]. Concurrently, Arabidopsis TGA protein sequences were sourced from the Arabidopsis Information Resource (TAIR) (https://www.arabidopsis.org (accessed on 1 October 2024)) [47]. To begin genome-wide identification, the “Blast Compare Two Segs” plug-in within TBtools (version 2.110) was utilized to input the protein sequence file of the Arabidopsis *TGA* gene family and the genome protein file for banana into their respective content boxes. The output file name was specified and “Table” was selected as the output format, then the process was initiated by clicking “start”. The resulting file was opened in Excel, where it was sorted and processed to identify the candidate gene IDs. In the subsequent step, the “Fasta Extract or Filter” function of TBtools (version 2.110) was employed to extract the protein sequences of the identified candidate genes. The first input box contained the protein sequence of the banana genome, the second box specified the output file name, and the third box included the candidate gene IDs obtained from the previous step, thereby retrieving the protein sequences of the candidate genes. Hidden Markov Models (HMMs) corresponding to the TGA protein domains (PF07716, PF14144) were obtained from the Pfam database (http://pfam-legacy.xfam.org/ (accessed on 3 October 2024)) and subsequently employed to query the banana protein database. This process yielded an initial list of potential candidate proteins [48,49]. The candidate protein sequences were examined for conserved domains utilizing the NCBI Conserved Domain Database (CDD, https://www.ncbi.nlm.nih.gov/cdd (accessed on 10 October 2024)). Redundant sequences were eliminated, resulting in the selection of the 18 *MaTGA* family members, which were designated as *MaTGA1* through *MaTGA18* according to their respective chromosomal locations [50]. The MaTGA protein sequences were analyzed using the ExPASy ProtParam tool (https://web.expasy.org/protparam/ (accessed on 11 October 2024)) to determine their physicochemical properties, such as amino acid length (AA), molecular weight (MW), isoelectric point (pI), instability index, aliphatic index, and the grand average of hydropathicity (GRAVY) [51]. Additionally, predictions of the subcellular localization were conducted utilizing the Cell-PLoc 2.0 online platform (http://www.csbio.sjtu.edu.cn/bioinf/Cell-PLoc-2/ (accessed on 11 October 2024)) [52].

### 4.3. Chromosomal Localization and Phylogenetic Tree Construction

Utilizing the genomic data for banana, a chromosomal localization map for the *MaTGA* genes was developed using TBtools software (version 2.110). To explore the MaTGA protein phylogenetic relationships, the TGA protein sequences for Arabidopsis and rice were downloaded from TAIR10 (https://www.arabidopsis.org/ (accessed on 16 October 2024)) and RGAP (v7.0) (http://rice.uga.edu/ (accessed on 16 October 2024)), respectively, and the TGA protein sequences for tomatoes (v3.0) and grapes (v4.0) were downloaded from the NCBI database (https://www.ncbi.nlm.nih.gov/ (accessed on 16 October 2024)). Based on the full-length protein sequences, the Jones-Taylor-Thornton (JTT) model in MEGA 6.0 was used to align the protein sequences. The phylogenetic tree was then constructed using the Neighbor-Joining method, with the Bootstrap test parameter set to 1000.

### 4.4. Analysis of the Conserved Motifs, Conserved Domains, Protein Structure, and cis-Elements in the TGA Genes

The conserved motifs within the MaTGA proteins were analyzed utilizing the MEME Suite (https://meme-suite.org/meme/ (accessed on 13 October 2024)). Further examination of the conserved domains in the MaTGA proteins was conducted using the NCBI Conserved Domain Database (CDD, https://www.ncbi.nlm.nih.gov/cdd (accessed on 10 October 2024)). Using the SOPMA online website (https://npsa.lyon.inserm.fr/cgibin/npsa_automat.pl?page=/NPSA/npsa_sopma.html (accessed on 15 October 2024)), secondary structure prediction analysis was performed for the MaTGA proteins. Additionally, TBtools facilitated the extraction of 2000 bp upstream sequences of the *MaTGA* genes, and the PlantCARE platform (http://bioinformatics.psb.ugent.be/webtools/plantcare/html/ (accessed on 21 October 2024)) was employed for the prediction of promoter cis-regulatory elements [53].

### 4.5. Analysis of Gene Replication and Collinearity in the MaTGA Genes

To conduct the analysis, the “One Step MCScanX” plugin within TBtools (version 2.110) was employed. The prepared genome files for banana and other multiple species (including rice, soybean, Arabidopsis, and tomato), along with the corresponding GFF files, were inputted. The output path was specified, and the CPU parameter for BlastP was set to 4, while other parameters were maintained at their default settings. Subsequently, the Ctl, GFF, and Collinearity files obtained were inputted into the “Dual Synteny Plot” plugin. Optionally, the ID of the *MaTGA* gene was entered into the “Gene List for Highlight” field. The default parameters of the plugin were used throughout the process. Finally, the “start” button was clicked to initiate the analysis. Genomic data for Arabidopsis, tomato, rice, and soybean were obtained from the NCBI database (https://www.ncbi.nlm.nih.gov/ (accessed on 16 October 2024)) [54].

### 4.6. Prediction of MaTGA Protein–Protein Interactions 

The protein sequences of the *MaTGA* genes were analyzed for protein–protein interactions using the STRING database (https://cn.string-db.org/ (accessed on 1 November 2024)) [55]. Subsequently, the predicted interaction data were downloaded and further processed using Cytoscape_v3.7.2 software to visualize and refine the interaction network [56]. Cytoscape parameters used were Border Width “2.0”, Label Font Size “30”, Shape “Circle”, Circle Size “100”, and Transparency “255”. Other parameters were kept as default.

### 4.7. qRT-PCR Analysis of MaTGA Expression Across Various Banana Tissue Types and Nitrogen Concentrations

The RNAprep Pure Polysaccharide Polyphenol Plant Total RNA Kit (Tiangen Biochemical Technology, Beijing, China) was employed to extract the total RNA from various banana tissues, including the roots, stems, leaves, flowers, and fruits, as well as from the banana roots subjected to different nitrogen levels (100% N, 70% N, 20% N, and 0% N) over a period of three days. The concentration and purity of the extracted total RNA were assessed using a NanoDrop Lite spectrophotometer (Thermo Fisher Scientific, Waltham, MA, USA), ensuring an OD260/280 ratio within the range of 1.8 to 2.0. Subsequently, the Evo M-MLV Reverse Transcription Premix Kit Version 2, which includes a genomic DNA removal reagent for quantitative PCR, was utilized to synthesize the complementary DNA (cDNA) from the total RNA for quantitative fluorescence analysis. Quantitative real-time PCR (qRT-PCR) primers were designed using the Batch qPCR Primer Design plug-in available in the TBtools software (Supplementary Table S1). The reagent specifications for the ChamQ Universal SYBR qPCR Master Mix, provided by Nanjing Nuovican Biotechnology Co., LTD, was consulted. Real-time fluorescence quantitative PCR was conducted using the qTOWER3G IVD instrument (Analytik Jena, Jena, Germany) under the following thermal cycling conditions: an initial denaturation at 95 °C for 30 s, followed by 40 cycles of 95 °C for 10 s and 56 °C for 30 s. The experiment was performed in triplicate using three biological replicates. The expression of the *MaTGA* genes was normalized to the internal reference gene *MaActin* in bananas, and data analysis was performed using the 2^−ΔΔCt^ method [57]. Statistical significance of the differences in gene expression levels was evaluated using SPSS software version 16.0 (using *p* < 0.05 as the threshold), and GraphPad Prism version 10 was employed to generate the histograms of gene expression.

## 5. Conclusions

In this study, a total of 18 *TGA* family members were identified within the banana genome. Comprehensive analyses were conducted on their physicochemical properties, phylogenetic relationships, gene structures, gene duplication events, promoter cis-acting elements, and protein interactions. The *MaTGA* family members were categorized into four distinct subfamilies, which are unevenly distributed across eight chromosomes. The TGA promoter sequences encompassed various cis-acting elements, including abscisic acid response elements (ABRE), ethylene response elements (ERE), salicylic acid response elements (SARE), in addition to drought response elements (MBS) and low temperature response elements (LTR). In terms of gene expression profiles, the expression levels of *MaTGA7*, *MaTGA8*, and *MaTGA15* were markedly elevated in the leaves and roots relative to other tissues. The expression levels of *MaTGA7*, *MaTGA8*, and the other 13 members were maximized under a nitrogen reduction treatment of 70%, followed by reduced expression at low nitrogen levels (20%), and were minimal under nitrogen-depleted conditions. These findings imply that the *MaTGA* genes may be integral to enhancing nitrogen use efficiency. Furthermore, protein–protein interaction predictions indicated that MaTGA7, MaTGA8, and MaTGA15 potentially interact with genes associated with nitrate transport and hormone regulation (such as NRT2.1, NRT2.2, NLP7, NPF6.3, TCP20, etc.), engaging in biological processes such as nitrogen absorption, transport, and assimilation. These findings establish a basis for future investigations into the potential roles and molecular regulatory mechanisms of the *MaTGA* genes in optimizing nutrient utilization and responding to abiotic stress in bananas.

## Figures and Tables

**Table 1:** Basic physical and chemical properties of the TGA proteins in bananas.

Gene ID	ReGene ID	Gene Location	AA	Molecular Weight (kDa)	Theoretical pI	Instability Index	Aliphatic Index	Grand Average of Hydropathicity	Predicted Location
Macma4_02_g03320.1	*MaTGA1*	chr02	476	52.07	6.44	57.12	82.08	−0.416	Nucleus.
Macma4_02_g04430.1	*MaTGA2*	chr02	499	54.93	6.29	47.18	76.73	−0.469	Nucleus.
Macma4_02_g10240.1	*MaTGA3*	chr02	469	51.98	5.83	66.84	76.63	−0.516	Nucleus.
Macma4_03_g11390.1	*MaTGA4*	chr03	441	49.08	6.61	65.27	75.74	−0.503	Nucleus.
Macma4_03_g25330.1	*MaTGA5*	chr03	467	51.88	6.44	50.97	82.59	−0.368	Nucleus.
Macma4_03_g27710.1	*MaTGA6*	chr03	332	37.05	8.73	56.57	82.41	−0.514	Nucleus.
Macma4_05_g20700.1	*MaTGA7*	chr05	376	41.96	7.07	56.37	80.24	−0.425	Nucleus.
Macma4_07_g21390.1	*MaTGA8*	chr07	396	44.82	5.98	48.62	77.65	−0.571	Nucleus.
Macma4_08_g05470.1	*MaTGA9*	chr08	332	37.06	7.79	57.01	81.23	−0.522	Nucleus.
Macma4_08_g23060.1	*MaTGA10*	chr08	467	50.92	9.06	51.65	73.23	−0.528	Nucleus.
Macma4_09_g15770.1	*MaTGA11*	chr09	429	47.37	8.38	57.95	86.97	−0.286	Nucleus.
Macma4_09_g19200.1	*MaTGA12*	chr09	483	54.31	8	63.68	79.05	−0.483	Nucleus.
Macma4_09_g29930.1	*MaTGA13*	chr09	445	49.94	8.5	68.15	75.96	−0.526	Nucleus.
Macma4_10_g08930.1	*MaTGA14*	chr10	509	56.69	8.85	58.43	78.11	−0.576	Nucleus.
Macma4_10_g34180.1	*MaTGA15*	chr10	375	42.82	8.69	48.24	79.63	−0.417	Nucleus.
Macma4_11_g13990.1	*MaTGA16*	chr11	504	56.11	6.56	52.71	73.23	−0.484	Nucleus.
Macma4_11_g24440.1	*MaTGA17*	chr11	448	49.38	7.08	61.04	78.71	−0.498	Nucleus.
Macma4_11_g25460.1	*MaTGA18*	chr11	484	53.33	6.77	50.37	76.12	−0.487	Nucleus.

**Table 2:** Secondary structure properties of the MaTGA protein family.

Protein	Proportion of Secondary Structure Elements (%)				Distribution of Secondary Structure Elements
	Alpha-Helix	Beta-Turn	Random Coil	Extended Strand	
MaTGA1	53.15%	1.68%	40.34%	4.83%	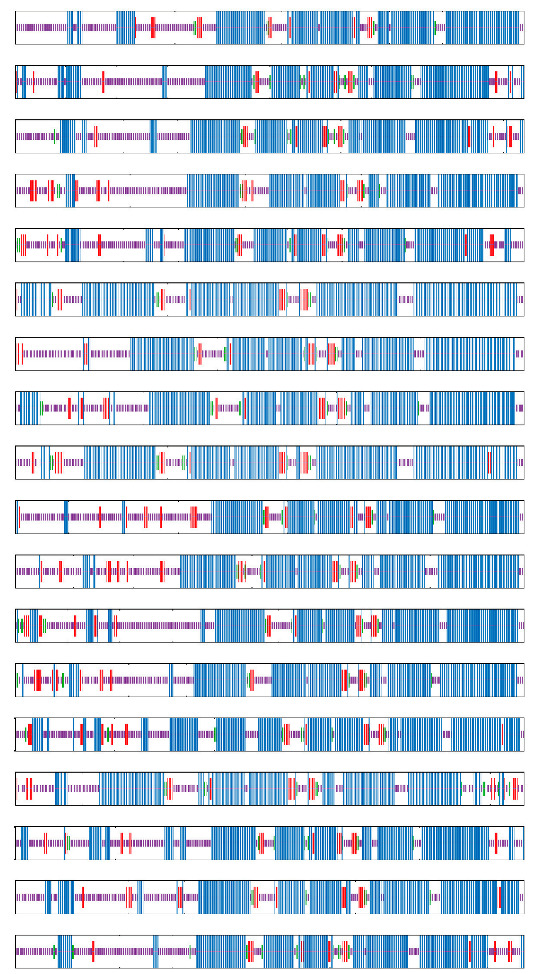
MaTGA2	50.70%	2.81%	41.08%	5.41%	
MaTGA3	52.67%	2.77%	38.59%	5.97%	
MaTGA4	52.38%	1.81%	36.96%	8.84%	
MaTGA5	51.82%	2.57%	38.12%	7.49%	
MaTGA6	74.70%	1.81%	17.17%	6.33%	
MaTGA7	58.78%	2.13%	32.45%	6.65%	
MaTGA8	60.86%	2.53%	30.30%	6.31%	
MaTGA9	69.58%	2.71%	19.58%	8.13%	
MaTGA10	50.54%	1.71%	40.69%	7.07%	
MaTGA11	53.85%	1.63%	35.90%	8.62%	
MaTGA12	51.97%	2.69%	38.72%	6.63%	
MaTGA13	53.93%	2.25%	36.18%	7.64%	
MaTGA14	55.80%	2.75%	34.58%	6.88%	
MaTGA15	57.33%	3.73%	30.40%	8.53%	
MaTGA16	51.98%	2.58%	39.68%	5.75%	
MaTGA17	54.91%	1.34%	37.28%	6.47%	
MaTGA18	49.79%	3.51%	41.12%	5.58%	

The proportion and distribution of the four structural elements were displayed. The green line represents β-turn, the blue line represents α-helix, the purple line represents random coil, and the red line represents extended strand.

## Data Availability

All the other data supporting the conclusions of this article are included within the article and its Supplementary Materials.

## Supplementary Materials

The following supporting information can be downloaded at: https://www.mdpi.com/article/10.3390/ijms26052168/s1.
