# Supplementary figures and images for: Genome-Wide Identification and Expression Analysis of the TGA Gene Family in Banana (Musa nana Lour.) Under Various Nitrogen Conditions
Source: Int J Mol Sci. 2025 Feb 28;26(5):2168. doi: 10.3390/ijms26052168 (PMC11900138; doi:10.3390/ijms26052168)

# Motif1

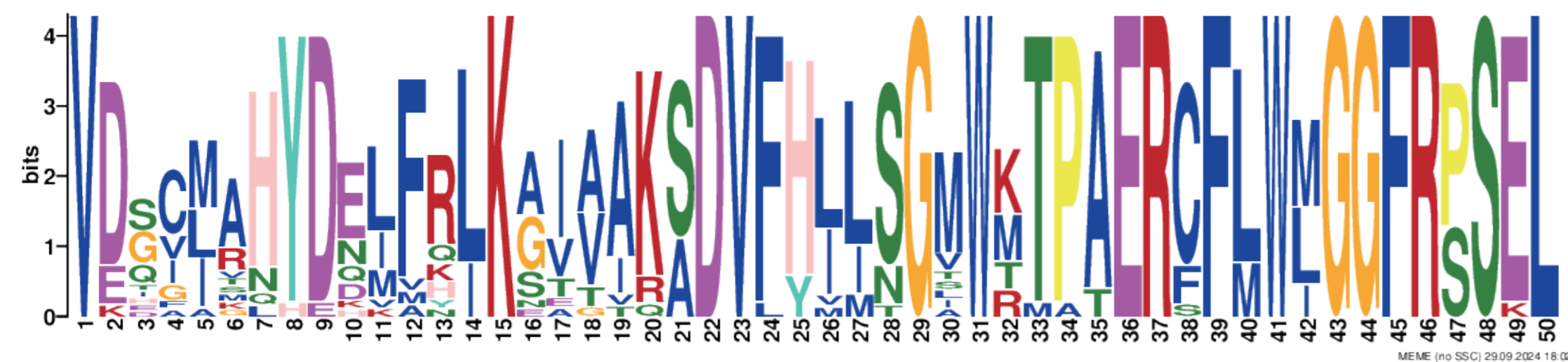

# Motif6

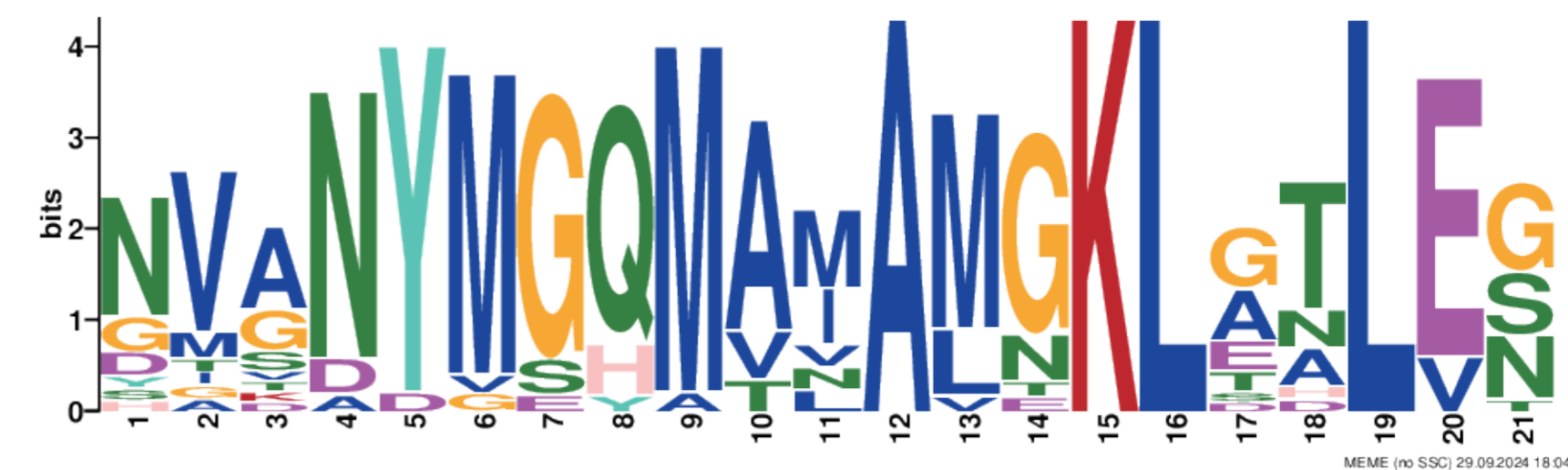

# Motif2

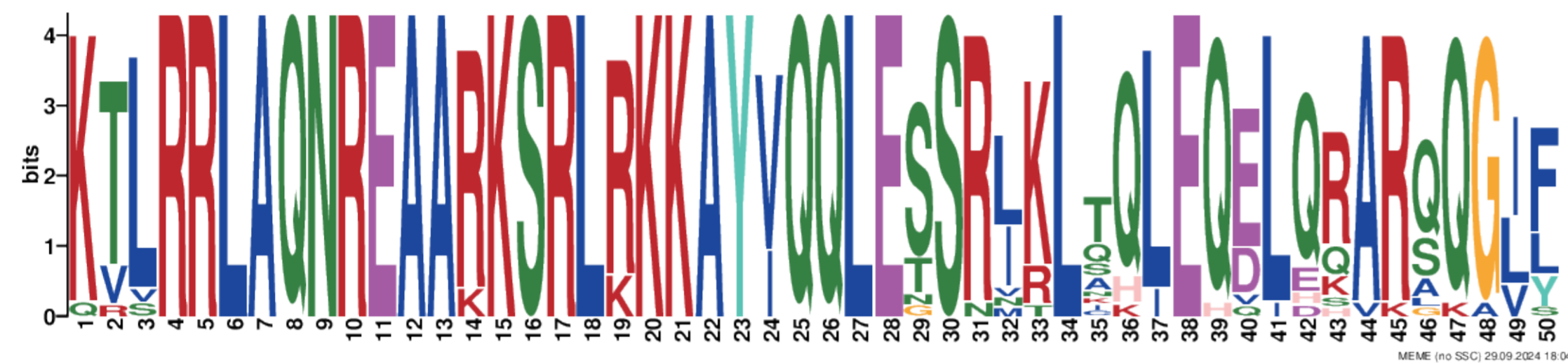

# Motif7

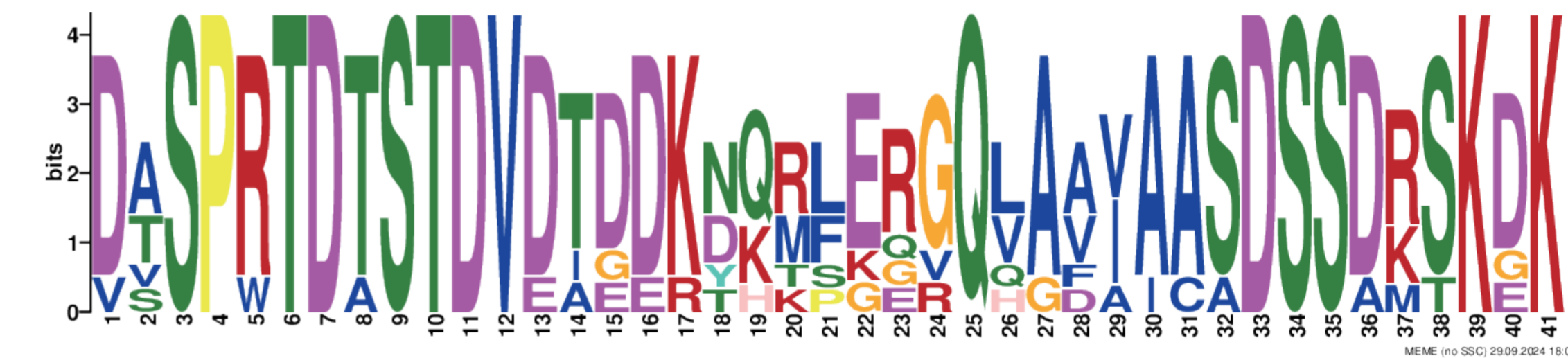

# Motif3

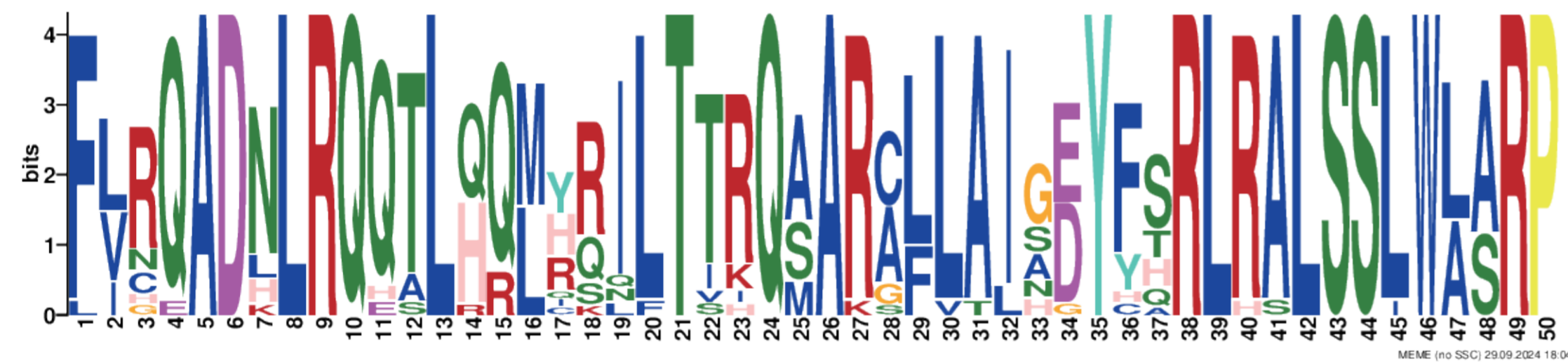

# Motif8

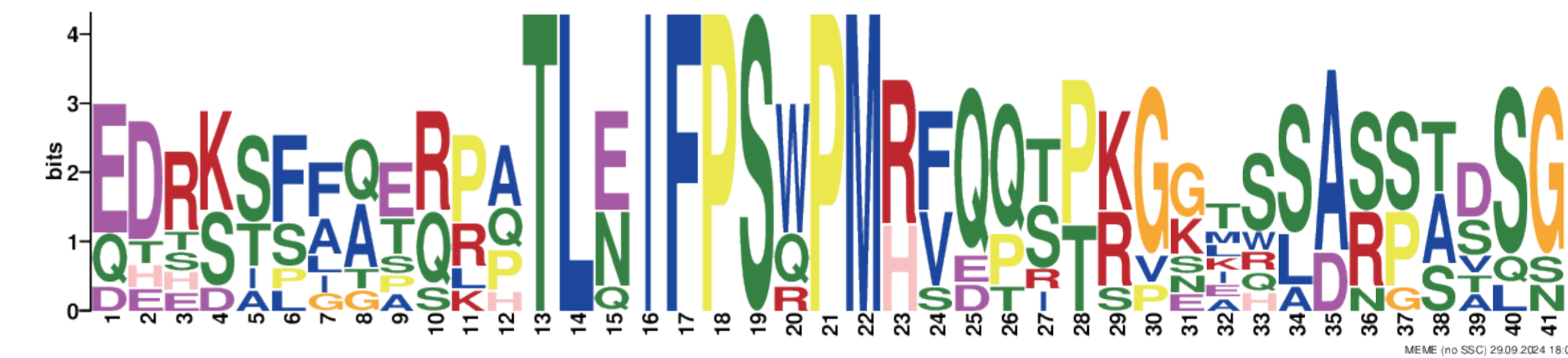

# Motif4

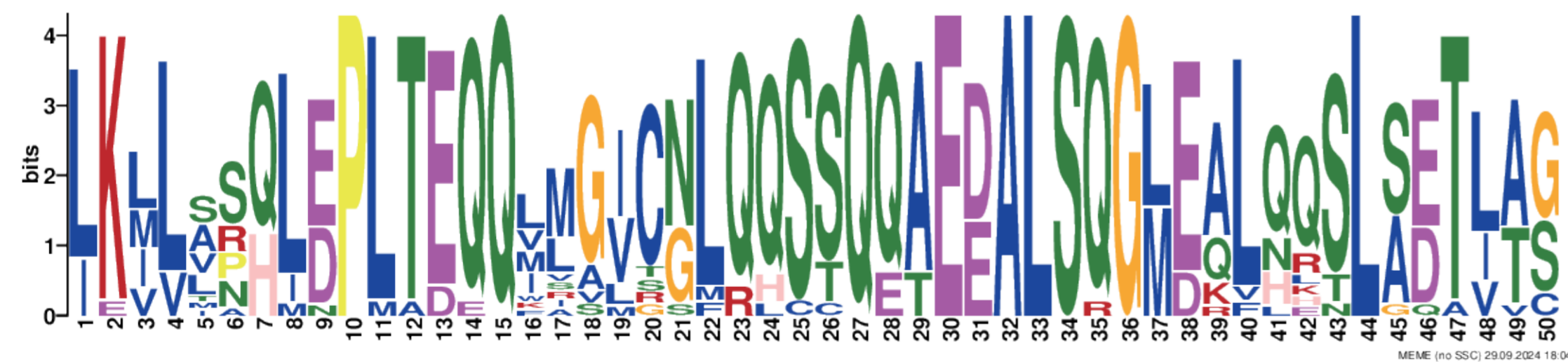

# Motif9

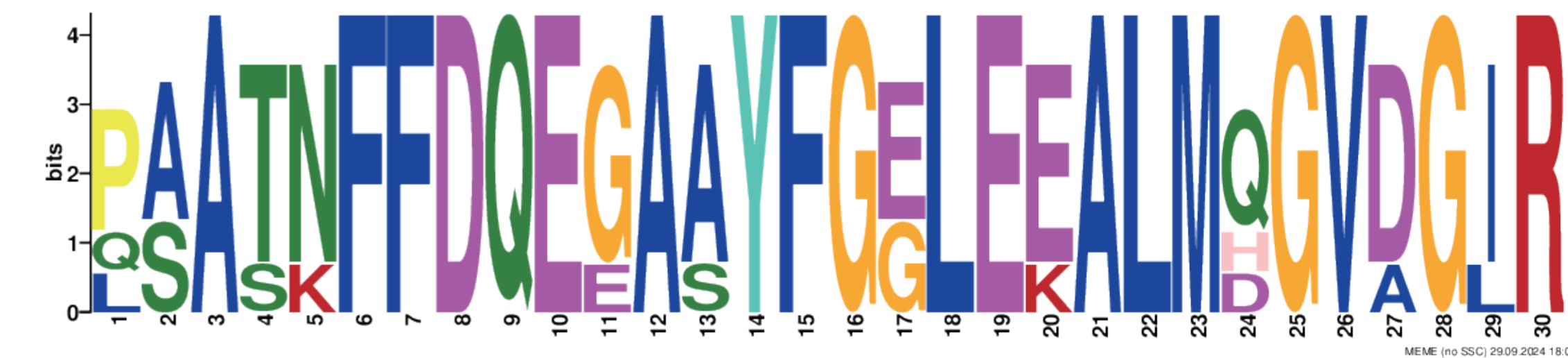

# Motif5

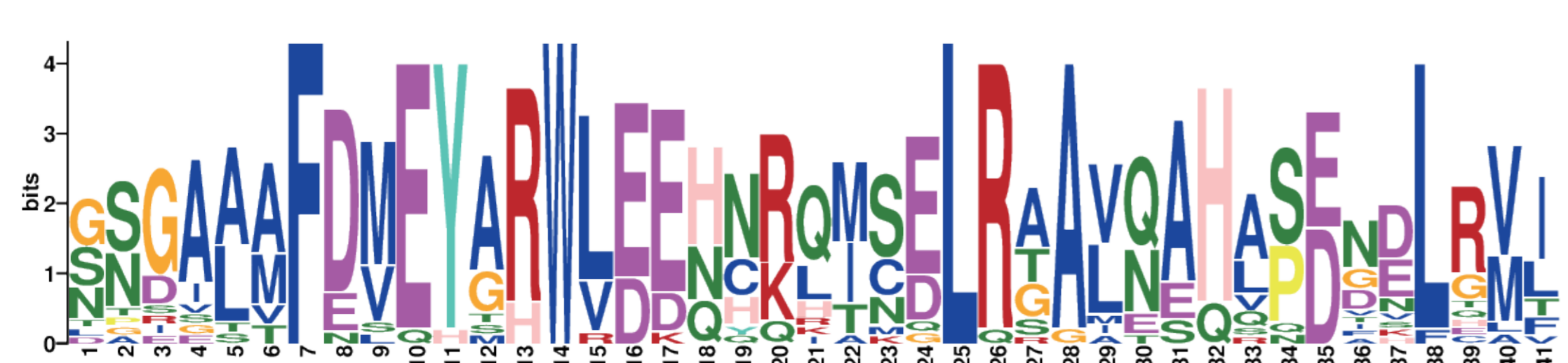

# Motif10

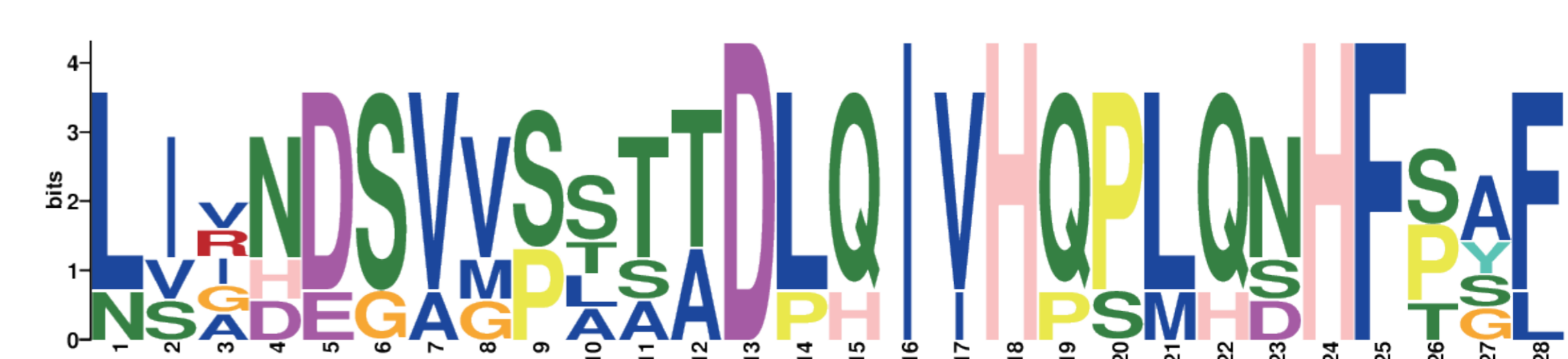

Supplement: Supplementary file 1 [file ijms-26-02168-s001.zip › Supplementary FigureS1:Motif identification information..pdf]

**A**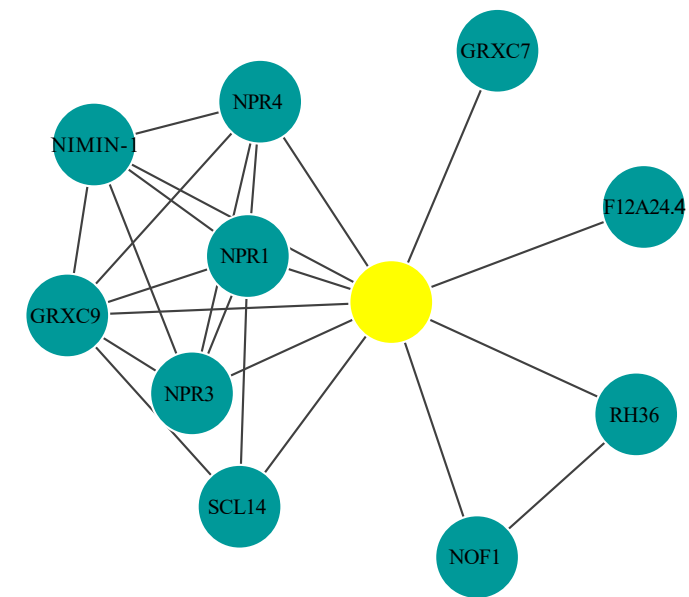

● MaTGA1, MaTGA2, MaTGA3, MaTGA5, MaTGA6, MaTGA9, MaTGA10, MaTGA11, MaTGA12, MaTGA14, MaTGA16, MaTGA17, MaTGA18

**B**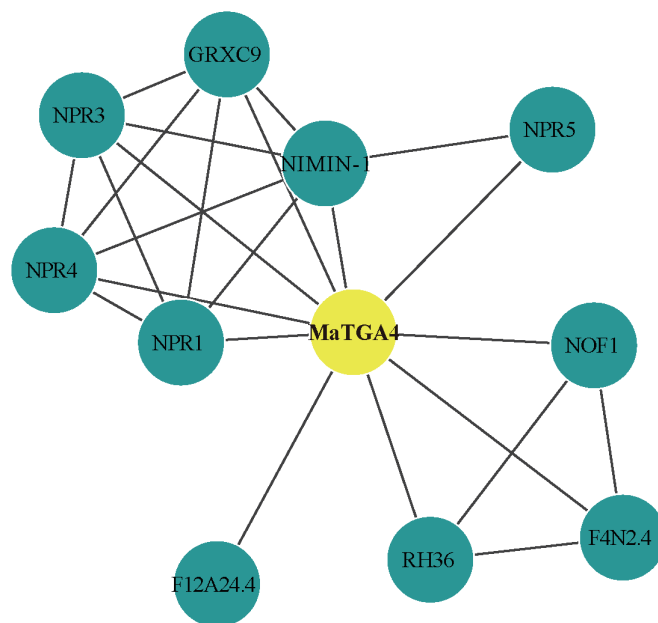**C**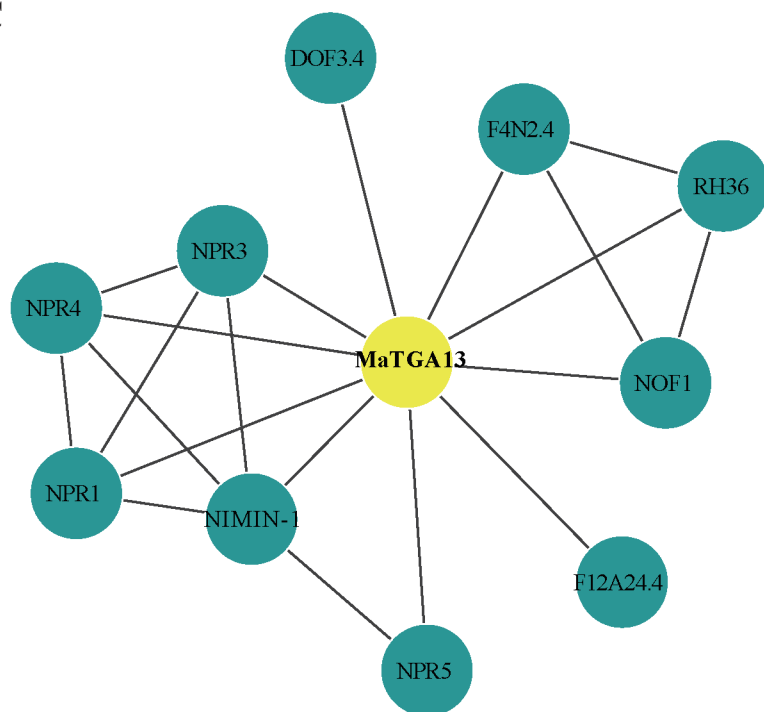**D**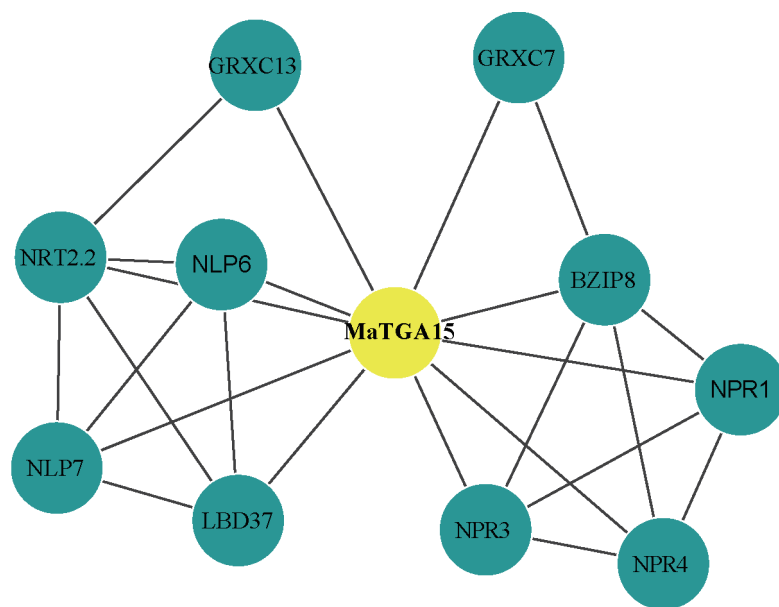

Supplement: Supplementary file 1 [file ijms-26-02168-s001.zip › Supplementary FigureS2:MaTGA proteins interaction prediction.pdf]
